# Propranolol-Induced Circulatory Collapse in a Patient With Thyroid Crisis and Underlying Thyrocardiac Disease: A Word of Caution

**DOI:** 10.1177/2324709617747903

**Published:** 2017-12-20

**Authors:** Hossam Abubakar, Vijendra Singh, Anandita Arora, Sammar Alsunaid

**Affiliations:** 1 Detroit Medical Center, Wayne State University, Detroit, MI, USA

**Keywords:** thyroid storm, heart failure, propranolol, endocrinology, cardiovascular collapse, adverse reaction

## Abstract

Thyrotoxic crisis or thyroid storm is a severe form of hyperthyroidism and a rare endocrinological emergency. The cornerstones of medical therapy in thyroid storm include decreasing the levels of circulating T3 in the blood as well as inhibiting the hormone’s peripheral effects through β-adrenergic blockade. Propranolol is the preferred agent for β-blockade in hyperthyroidism and thyroid storm due to its additional effect of blocking the peripheral conversion of inactive T4 to active form T3. We report a typical clinical scenario where propranolol was administered in treatment of thyroid storm but an uncommon adverse outcome: circulatory failure from cardiogenic shock warranting vasopressor and inotropic support. Caution with regard to the use long-acting β-blocking agents in patients with underling thyrocardiac disease may prevent this life-threatening adverse effect. Ultra–short-acting β-blockers that are easy to titrate maybe a suitable alternative in this subset of patients.

## Background

Thyrotoxic crisis or thyroid storm is a severe form of hyperthyroidism and a rare endocrinological emergency. The cornerstones of medical therapy in thyroid storm include decreasing the levels of circulating T3 in the blood as well as inhibiting the hormone’s peripheral effects through β-adrenergic blockade. Propranolol is the preferred agent for β-blockade in hyperthyroidism and thyroid storm due to its additional effect of blocking the peripheral conversion of inactive T4 to active form T3. Patients presenting with thyroid storm may have clinical or subclinical thyrocardiac disease that may predispose them to an exaggerated response to β-blocker therapy manifesting as circulatory collapse secondary to cardiogenic shock.

## Case Presentation

A 39-year-old African American man with known hyperthyroidism, atrial fibrillation, and systolic heart failure presented to the emergency room with complaints of dyspnea, chest pain, palpitations, nausea, vomiting, and worsening pedal edema. Since his diagnosis 5 years ago, he has had multiple admissions for thyrotoxicosis secondary to medication nonadherence.

**Figure 1.**
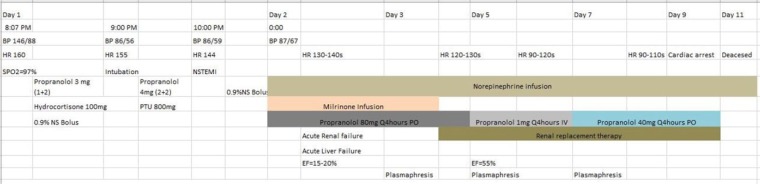
Electrocardiogram done on presentation to emergency room, showing ventricular rate of 140 beat per minute and atrial flutter with 2:1 atrioventricular conduction.

**Figure 2.**
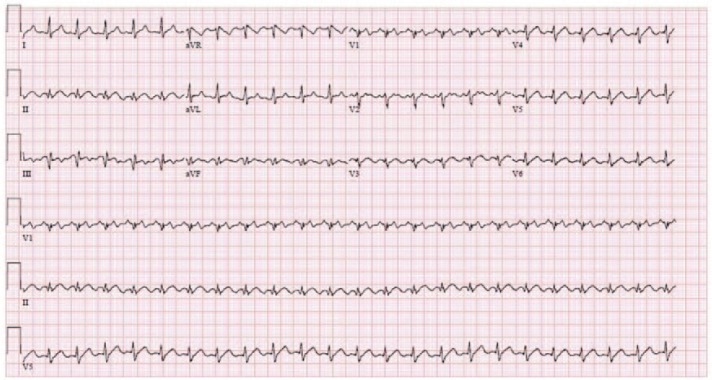
Events timeline.

On admission (at 8:07 pm), he was tachycardic with a heart rate (HR) of 160 beats per minute (bpm) and hypertensive with a blood pressure (BP) of 146/89 mm Hg. Electrocardiogram revealed atrial flutter with 2:1 atrioventricular block (Figure 1). Basic metabolic panel and complete blood count results were within normal limits. A clinical diagnosis of thyroid storm was made using Burch and Wartofsky’s criteria. Our patient scored 55 points on the Burch-Wartofsky point scale (10 points: the presence of an arrhythmia; 10 points: presence of a precipitant [medication nonadherence]; 5 points: pedal edema; 25 points: HR > 140 bpm; 5 points: temperature of 37.2°C to 37.7°C). A score of 55 is highly suggestive of a diagnosis of a thyroid crisis (Figure 2).

Intravenous (IV) propranolol (1 mg at 8:17 pm and 2 mg at 8:24 pm), hydrocortisone (100 mg at 8:28 pm), and propylthiouracil (PTU; 800 mg oral at 9 pm) were administered. He subsequently developed respiratory distress and was intubated (8:45 pm). An hour later, he became hypotensive with a BP 86/56 mm Hg (9:00 pm). Administration of 1 liter of IV normal saline bolus led to an improvement of BP to 92/65 mm Hg. Oral iodine (10 drops lugols solution at 10 pm) was also given.

Further laboratory studies revealed a free thyroxine (FT4) level of 4.1 ng/dL (normal = 0.8-1.8 ng/dL), thyroid-stimulating hormone level of <0.008 µIU/mL (normal = 0.20-4.78 µIU/mL), troponin I of 0.533 ng/mL (normal high = 0.057 ng/mL), and a lactic acid of 11.3 mmol/L (normal = 0.4-2 mmol/L). Chest X-ray showed a right lower lobe airspace opacity. Two hours after administration of propranolol (7 mg IV and 80 mg PO), he became hypotensive again at 86/59 mm Hg and had persistent tachycardia of 144 bpm (10 pm). A 2-liter bolus of IV normal saline was administered with no improvement in BP. Consequently, norepinephrine (1-2 µg/kg/min titrated to achieve a mean arterial pressure [MAP] of >65 mm Hg) along with broad-spectrum antibiotics; vancomycin and cefepime was started (1 am on day 2). Repeat laboratory measurement revealed further increase in serum troponin level at 15.10 ng/mL. Electrocardiogram showed T wave inversions in leads V1 to V4, and IV heparin was consequently started for treatment of non-ST segment myocardial infarction. Propranolol at 80 mg every 4 hours through a nasogastric tube was continued along with norepinephrine due to the patient’s persistent tachycardia.

Repeat laboratory measurements on day 2 of hospitalization revealed acute liver failure and anuric acute renal failure. PTU was consequently stopped due to liver injury (only dose was administered). Transthoracic echocardiography (TTE) showed 15% to 20% ejection fraction (EF), global hypokinesia, and torrential tricuspid regurgitation. Milrinone IV infusion was added to norepinephrine (on day 2 at 11:14 am) for inotropic support due to the patient’s markedly reduced EF. On the third day of hospitalization, he underwent plasmapheresis for refractory thyroid storm after which his HR and BP showed improvement (HR = 120-130 bpm and BP = 107/55). Consequently, milrinone was discontinued and norepinephrine dosage was down-titrated from 0.1to 0.05 µg/kg/min achieving a MAP of greater than 65 mm Hg. Repeat TTE on day 5 of hospitalization showed normal EF and mild tricuspid regurgitation. Acute renal failure was treated with continuous renal replacement therapy. The patient underwent 2 subsequent plasmapheresis sessions (on days 5 and 7 of hospitalization) with further improvement in BP at 100-110/70-80 mm Hg. Despite the initial improvement, the patient underwent an episode of cardiac arrest with pulseless electrical activity on day 9 of hospitalization. Cardiopulmonary resuscitation was initiated along with 1 mg of IV epinephrine every 4 minutes and a return of spontaneous circulation was achieved after a total 21 minutes. His condition continued to worsen over the next 2 days and a shared decision was made to stop further escalation of his care by the patient’s family and medical team. The patient died on day 11 of hospitalization.

## Discussion

Hyperthyroidism affects the cardiovascular system in several and complex ways.^1^ These effects are mediated by induction of thyroid nuclear receptors resulting in gene transcription or by the hormone’s direct effect on extranuclear cell components.^2^ These lead to the combined effects of increased contractility, improved diastolic relaxation, and decreased peripheral resistance creating a high cardiac output (CO) state. In the resting state, this allows hyperthyroid patients to compensate for the abnormally increased metabolic demand through increased CO. However, during stress states, for example, exercise, there is a failure to increase CO further to meet the superimposed exercise-induced increase in metabolic demand, a phenomenon known as reduced contractile reserve.^3^ The manifestations of heart failure (HF) symptoms in a setting of high CO have been attributed to the hyperthyroid-associated decrease in contractile reserve.^4^ This has been commonly identified as “high-output” HF. Many have argued that that the term is inaccurate,^5^ as the CO remains elevated during both the resting and stress states and the decompensation is functional, that is, not associated with failure or cessation of the high output state.^4^ Furthermore, although less common, prolonged severe hyperthyroidism may lead to HF with reduced CO. The cause of low-output failure in hyperthyroid patients is likely multifactorial.^5^ Among the causes is persistent tachycardia in poorly controlled hyperthyroid patients causing ventricular dilation and biventricular systolic failure.^6^ Other risk factors include preexisting hypertension, valvular, and ischemic heart disease.^7^ Additionally, the pathologic increase in cardiac workload in hyperthyroid states can occasionally surpass the compensatory capacity of coronary vasodilation and induce episodes of ischemia and consequent systolic dysfunction.^8^

Thyrotoxic crisis or thyroid storm is a severe form of hyperthyroidism and a rare endocrinological emergency with a mortality rate of 10% to 30%.^9^ The pathophysiology associated is complex and includes an amplified response to T3 and an abrupt increase in the levels of free hormone attributed to a decrease in protein carrier capacity.^10^ Treatment is multimodal including medical, surgical, and supportive care.^9^ The main cornerstones of medical therapy include decreasing the levels of circulating T3 in the blood as well as inhibiting the hormone’s peripheral effects through β-adrenergic blockade.^9^

Hyperthyroidism induces a hyperadrenergic state characterized by an exaggerated sensitivity to circulating catecholamines.^2^ This is achieved by the hormone’s ability to increase β-adrenergic receptor density through the amplified formation and reduced degradation.^11^ In an attempt to impede this hyperadrenergic state, non–cardio-selective β-blockers (NCBB) have been used widely as the standard of therapy in both thyrotoxic crisis and uncomplicated hyperthyroidism.^12^ Propranolol has been a preferred NCBB due to its additional effect of blocking the peripheral conversion of inactive T4 to active form T3.^9^ Our case demonstrates a typical clinical scenario where propranolol was administered in the treatment of thyroid storm but an uncommon adverse outcome: circulatory failure from cardiogenic shock warranting vasopressor and inotropic support. Our presented patient suffered a severe drop in MAP shortly following administration of IV propranolol. TTE done after decompensation revealed a low left ventricular ejection fraction (LVEF) of 10% to 15%, which was a decrease from a previously known LVEF of 25% to 30% seen on TTE done 2 months prior. The temporal association of IV propranolol administration and the severe drop in MAP and LVEF led us to conclude that it was the cause or at least trigger of the hemodynamic decompensation and cardiogenic shock. Through a systematic literature search of 2 databases (PubMed and Embase), we allocated 9 published reports (7 full-text articles and 2 conference abstracts) of similar cases of β-blockade-induced cardiovascular collapse in patients with thyroid storm (Table 1).

**Table 1.** Reported Cases of β-Blocker–Induced Circulatory Collapse in Patients With Thyroid Storm.

Study	Patient	Thyroid Disease	Evidence of HF^a^	β-Blocker and Dose	Type of Circulatory Collapse	Post–β-Blockade TTE	Hospital Course After Episode of Circulatory Collapse
Yamashita et al^16^ (2015)	62, female	Grave’s disease; thyroid storm	LVEF of 30%	Bisoprolol (dose not reported)	Hypotensive	Not reported	Bisoprolol was discontinued after the hypotensive episode and the patient remained tachycardic. Lindolol chloride was initiated for HR control and did not cause further drops in SBP. HR was successfully controlled and TTE after stabilization of HR showed LVEF of 55%. Tachycardia recurred and patient underwent thyroidectomy after which was discharged home in stable condition.
Vijayakumar et al^17^ (1989)	85, female	Multinodular goiter; thyroid storm	History of HF (details not reported)	Propranolol 4 mg IV, 20 mg PO	Hypotensive with atrial fibrillation	Not reported	Resuscitation with, atropine, adrenaline, and dobutamine was initiated. Persistent AF and tachycardia after propranolol discontinuation was accompanied by acute limb ischemia. Esmolol infusion was initiated and carefully titrated for HR control and dobutamine was continued for BP support. 36 hours later, the patient was hemodynamically stable and underwent total thyroidectomy and right-sided AKA and was discharged in stable condition.
Ngo and Tan^20^ (2006)	32, male	Grave’s disease; thyroid Storm	CXR shows cardiomegaly with mild congestion	Propranolol 10 mg PO	Hypotensive with atrial flutter	LVEF of 25% with severe TR and MR	No further dose of propranolol was administered. Patient underwent successful cardioversion for atrial flutter and was put on inotropic support along with intra-aortic balloon pump. Patient stabilized after resuscitative measures and was discharged in stable condition.
Narechania et al^21^ (2015)	27, female	Grave’s disease; thyroid Storm	CXR shows cardiomegaly	Metoprolol (dose not reported)	Cardiac arrest (PEA)	Global LV dysfunction with severe MR and TR	Successful resuscitation with chest compressions and epinephrine (no further details reported).
Eleftheriou et al^22^ (2010)	39, female	Thyroid storm	LVEF of 35%	Propranolol 2 mg IV	Cardiac arrest	LVEF of 15%	CPR initiated with no response. Diagnosis of cardiogenic shock was made and attempt of Extracorporeal cardiovascular support with ECMO was not successful. Patient subsequently developed multi-organ failure and expired 5 days later.
Fraser et al^23^ (2001)	52, female	Thyroid storm	LVEF of 35%	Sotalol 1 mg/kg IV	Cardiac arrest	Global impairment of LV function	CPR initiated with successful return of pulse. Patient remained hypotensive requiring vasopressor and inotropic support to maintain blood pressures. 24 hours later patient was hemodynamically stable and was discharged on day 10 of admission. Repeat TTE 6 weeks later revealed LVEF within normal range.
Boccalandro et al^24^ (2003)	48, female	Grave’s disease; thyroid storm	S3, JVD, bilateral crackles, hepatomegaly, hepatojugular reflex	Propranolol 40 mg PO	Hypotensive	LVEF <20%, 4-chamber dilation, MR and TR	Supportive care with hemodynamic stability within 24 hours (no further details reported).
Ashikaga et al^25^ (2000)	50, female	Grave’s disease; thyroid storm	Not reported	Propranolol (dose not reported)	Hypotensive	Four-chamber dilation with reduced LV function	Propranolol was D/C. Vasopressor and inotropic therapy were initiated with successful hemodynamic stabilization and improvement in cardiac index. Predischarge TTE was normal. Patient discharged in stable condition.
Dalan and Leow^15^ (2007)	43, female	Thyroid storm	No reported evidence	Propranolol 20 mg PO	Cardiac arrest (asystole)	LVEF 60% with dilated atria	Propranolol was D/C. CPR with successful return of pulse. Vasopressor initiated for BP control and patient recovered (no further details reported).
	43, female	Thyroid storm	LVEF of 45%, atrial dilation, TR	Propranolol 20 mg PO	Cardiac arrest (asystole)	Not reported	Propranolol therapy D/C. CPR and vasopressor support with initial improvement. Patient developed a second episode of cardiac arrest and consequently expired.

Abbreviations: HF, heart failure; TTE, transthoracic echocardiography; LVEF, left ventricular ejection fraction; HR, heart rate; SBP, systolic blood pressure; IV, intravenous; PO, oral; AF, atrial fibrillation; D/C, discontinued; BP, blood pressure; AKA, above knee amputation; CXR, chest X-ray; TR, tricuspid regurgitation; MR, mitral regurgitation; PEA, pulseless electrical activity; LV, left ventricle; CPR, cardiopulmonary resuscitation; ECMO, extracorporeal membrane oxygenation; JVD, jugular venous distention.

a Clinical, radiographic, or echocardiographic evidence of heart failure.

It is well proven that in patients with hyperthyroidism, increase in HR, contractility, and CO are a result of the direct effect of T3 and to a lesser extent due to the thyroid-induced hyperadrenergic state.^2^ This explains why in most patients with hyperthyroidism and thyroid crisis without HF, β-blocker therapy does bring symptomatic relief but does not cause a significant decrease in CO.^13^ On the contrary, it is suggested that in patients with hyperthyroidism and low-output cardiac failure, the thyroid-induced hyperadrenergic state plays a compensatory role in maintaining CO. Administration of β-blocker in this circumstance may halt this compensatory mechanism and cause a significant fall in CO and consequent hemodynamic instability.^14^ This is supported by all but one^15^ of the published case reports (Table 1), where patients had either clinical or echocardiographic evidence of HF when presenting with thyroid crisis. Our patient had a low LVEF of 35% and which was in parallel with the majority of published cases. In the case reported by Dalan et al,^15^ there was no clinical evidence of HF at presentation, and yet the patient developed cardiac arrest shortly after administration of propranolol. This raises the concern that patients with hyperthyroidism may harbor subclinical cardiomyopathy, which may also put them at high risk of the exaggerated sensitivity to β-blockade and resultant hemodynamic instability. Furthermore, the upregulation of β-adrenergic receptor density due to the effect of T3^11^ might have also contributed to the exaggerated response to β-adrenergic blockade evidenced by severe hypotension after propranolol administration. It is also interesting to note that in our case and 2 of the reported cases,^16,17^ the resultant hemodynamic instability was accompanied by uncontrolled tachycardia, which warranted continuation of β-blocker therapy. In the case by Vijayakumar et al,^17^ cessation of propranolol therapy was accompanied by persistent atrial fibrillation and uncontrolled tachycardia that was complicated by acute limb ischemia secondary to thromboembolism. This necessitated the initiation of esmolol, an ultra–short-acting NCBB for HR control. In the case reported by Yamashita et al,^16^ bisporol was discontinued after the hypotensive episode, and IV landiolol hydrochloride infusion was initiated for HR control. Landiol hydrochloride is an ultra–short-acting cardioselective β-blocker with a half-life of 4 minutes (similar to that of esmolol).^18^ In both cases, the doses of ultra–short-acting β-blockers were carefully titrated to the patients’ heart rates and resulted in successful rate control without hypotension. These cases demonstrate that complete cessation of β-blocker therapy may not be possible in hyperthyroid patients due to the uncontrolled tachycardia. Ultra–short-acting agents may be advantageous over long-acting NCBBs, for example, propranolol, and maybe a reasonable alternative in hyperthyroid patients and underlying thyrocardiac disease. This is due to their short half-lives, allowing for easier dose titration and rapid cessation of β-blocking effect after discontinuation of therapy,^19^ which may be necessary in the setting of circulatory collapse.

We believe that high awareness of this potential adverse effect is crucial because the use of NCBB in the treatment of thyroid crisis is the standard of care. Caution with regard to use in patients with underlying thyrocardiac disease may prevent life-threatening adverse events. Observing for symptoms and signs of HF and screening for subclinical cardiomyopathy using TTE maybe a reasonable approach to identifying those at risk of β-blocker–induced circulatory collapse. The use of easily titratable ultra–short-acing BB along with close hemodynamic monitoring and prompt discontinuation when appropriate may be a safer alternative for the widely used long-acting β-blocking agents.
